# Natural variation in Arabidopsis shoot branching plasticity in response to nitrate supply affects fitness

**DOI:** 10.1371/journal.pgen.1008366

**Published:** 2019-09-20

**Authors:** Maaike de Jong, Hugo Tavares, Raj K. Pasam, Rebecca Butler, Sally Ward, Gilu George, Charles W. Melnyk, Richard Challis, Paula X. Kover, Ottoline Leyser

**Affiliations:** 1 Sainsbury Laboratory, University of Cambridge, Cambridge, United Kingdom; 2 Department of Biology, University of York, York, United Kingdom; 3 Department of Biology and Biochemistry, University of Bath, Claverton Down, Bath, United Kingdom; University of Washington, UNITED STATES

## Abstract

The capacity of organisms to tune their development in response to environmental cues is pervasive in nature. This phenotypic plasticity is particularly striking in plants, enabled by their modular and continuous development. A good example is the activation of lateral shoot branches in Arabidopsis, which develop from axillary meristems at the base of leaves. The activity and elongation of lateral shoots depends on the integration of many signals both external (e.g. light, nutrient supply) and internal (e.g. the phytohormones auxin, strigolactone and cytokinin). Here, we characterise natural variation in plasticity of shoot branching in response to nitrate supply using two diverse panels of Arabidopsis lines. We find extensive variation in nitrate sensitivity across these lines, suggesting a genetic basis for variation in branching plasticity. High plasticity is associated with extreme branching phenotypes such that lines with the most branches on high nitrate have the fewest under nitrate deficient conditions. Conversely, low plasticity is associated with a constitutively moderate level of branching. Furthermore, variation in plasticity is associated with alternative life histories with the low plasticity lines flowering significantly earlier than high plasticity lines. In Arabidopsis, branching is highly correlated with fruit yield, and thus low plasticity lines produce more fruit than high plasticity lines under nitrate deficient conditions, whereas highly plastic lines produce more fruit under high nitrate conditions. Low and high plasticity, associated with early and late flowering respectively, can therefore be interpreted alternative escape vs mitigate strategies to low N environments. The genetic architecture of these traits appears to be highly complex, with only a small proportion of the estimated genetic variance detected in association mapping.

## Author summary

Many organisms adjust their development depending on environmental conditions. This is particularly striking in plants, with development constantly tuned throughout their lives. A good example is the modulation of shoot branching in response to external environmental cues. We characterised hundreds of genetically distinct *Arabidopsis thaliana* lines for their branching response to the availability of a key nutrient, nitrogen. We found that some lines adjusted their degree of branching according to the level of nitrogen available, whereas others did not. These latter low plasticity genotypes constitutively produce an intermediate number of branches and also flower earlier than the more plastic genotypes that responded to the nutrient treatment. One interpretation of these results is that flowering time and branch number are traded off in some way, reflecting at the extremes alternative strategies to cope with low nitrogen availability: an escape strategy of early flowering, and a mitigation strategy involving additional nutrient foraging. Using quantitative genetics methods, we found that the genetic basis for this response is likely to be complex, although not intractable when analysis methods that take into account genetic variability at the relevant candidate loci are used.

## Introduction

Most organisms experience environmental heterogeneity. For mobile organisms, adverse environments can be avoided by migration and habitat selection. Immobile organisms, such as higher plants, can avoid adverse environments by early reproduction and survival as seeds, or they may mitigate negative environmental impacts by physiological and/or developmental adjustments. It is therefore not surprising that plant development is so remarkably plastic, with a single plant genotype able to give rise to a wide range of phenotypes, depending on the prevailing environmental conditions [1].

There is a substantial body of theory, with some experimental support, concerning the circumstances under which plasticity is adaptive [2,3]. Key factors include the temporal and spatial scales of heterogeneity in the environment, which affect how well future conditions can be predicted from current environmental cues. For example, environments that are highly stable and therefore highly predictable, or environments that change too rapidly and stochastically relative to developmental responses for robust future prediction, may favour phenotypic canalisation. In contrast slower and more predictable environmental variation may favour plasticity [4]. Therefore, the question of whether or not plasticity is adaptive in nature is a complex one, the answer to which depends on the nature of the trait and its relationship with fitness, the costs associated with being plastic, the frequency and predictability of changes in the environment, and the amount of genetic variation for plasticity in populations [3,5,6]. In this context, it is interesting that closely related species in the plant kingdom can show widely differing degrees of phenotypic plasticity [4,7–11]. Quantifying the genetic variation in the plastic responses of a species and how it relates to fitness traits may help in understanding the ecological and adaptive significance of phenotypic plasticity more broadly.

Here, we use the annual model species *Arabidopsis thaliana* (Brassicaceae) as a system to dissect the genetics of shoot branching plasticity in response to nitrate. Arabidopsis is an ideal system for the study of plasticity. Firstly, the availability of many inbred lines from a wide geographic range provides ample genetic material for quantitative genetic studies [12]. Secondly, because these lines are highly inbred, they facilitate the study of plasticity at the genotype-level (rather than at the population-level), since the same genotypes can be grown in different environments. Because Arabidopsis is a natural selfer with high levels of inbreeding in wild populations, trait variance and covariance estimates from empirical studies should be less susceptible to changes due to artificial inbreeding as is seen in other model systems [13,14]. Finally, several studies in this species revealed substantial genetic variation in plasticity for traits such as flowering time, height, shoot branching and silique number in different growth conditions [15–23].

We focus on the plasticity of shoot branching and its relationship with other morphological and life-history traits. The major determinant of branch number, and particularly its plasticity, is the degree of activity of axillary shoot apical meristems, laid down in the axil of each leaf as they form on the primary shoot apical meristem. Shoot apical meristems can remain dormant, restricting the shoot system to a single axis of growth, or they can activate to produce a branch, reiterating the development of the primary axis, and allowing the possibility of higher order branches.

Axillary meristem activity is regulated by diverse inputs, including environmental factors such as nutrient availability, shading, and damage to the primary shoot apical meristem [24]. In addition, developmental inputs such as the position of an axillary meristem along the primary axis and the phase of growth of the plant (e.g. vegetative vs floral) have a profound effect on their activity. These inputs must be integrated to deliver an overall branching habit tuned according to the plant’s local environment, and there is compelling evidence that plant hormones are central to this integration. In Arabidopsis, a network of at least three interacting hormones—auxin, cytokinin and strigolactone—is required for the shoot branching response to nitrate supply [25,26]. Wild-type (Col) Arabidopsis plants grown under nitrate sufficient conditions produce more branches than those grown under nitrate deficient conditions. This is associated with a higher root biomass fraction in nitrate deficient conditions, a trait presumably associated with nitrate foraging [27]. Strikingly, plants deficient in cytokinin synthesis or signaling constitutively adopt branching levels similar to those of wild-type plants on low nitrate, whereas plants deficient in strigolactone synthesis or signaling constitutively adopt a high branching phenotype [25,26].

Thus, plasticity in response to nitrate supply depends on the hormone network, with constitutively extreme phenotypes associated with either low cytokinin or low strigolactone. This would suggest a mechanism for variation in branching plasticity in nature in which plants with low plasticity adopt these extreme phenotypes due to compromised strigolactone or cytokinin biology. However, the roles of these hormones in shoot branching have largely been elucidated using null alleles, which simultaneously affect both the activities of the genes in question and any ability to modulate these activities dynamically in response to environmental cues, making it difficult to assess their likely roles in variation in plasticity in nature.

To address whether and how plasticity varies in natural genotypes, we have analysed shoot branching responses to nitrate supply in two populations of Arabidopsis: a set of recombinant inbred lines from a mapping population derived from 19 accessions (the MAGIC lines [28]) and a set of natural accessions for which genome-wide genotype data are available [29–31]. Our results show significant natural variation in shoot branching plasticity in response to nitrate in both populations. We show that this plasticity correlates strongly with flowering time and has contrasting effects on fruit set depending on the available nitrate. This is consistent with a continuum of responses to N limitation, with escape through early flowering and mitigation through nitrogen foraging at the extremes. These traits are genetically complex, likely due partially to allelic heterogeneity at the relevant loci.

## Results

### Extensive natural variation in shoot branching response to nitrate supply

To investigate whether there is natural genetic variation for shoot branching plasticity in response to nitrate supply, we analysed a set of 374 Multi-parent Advanced Generation Inter-Cross (MAGIC) Arabidopsis lines grown under nitrate sufficient (high N—HN) and nitrate deficient (low N—LN) conditions [25,28]. Plants were monitored daily and the flowering time of each plant recorded. When the first two siliques were full, we scored the height of the main inflorescence and total number of primary branches (shoots > = 1cm). Four to eight plants from each genotype were scored in each condition (median n = 8), allowing us to assess how much of the variation in each trait was due to genetic and/or non-genetic effects. We partitioned the variance of each trait using linear mixed models that included terms accounting for differences between genotypes (genetic effects), differences between N treatments (environmental effects), and genetic differences in the degree of response to N supply (genotype-by-environment, GxE, interaction).

**Fig 1:**
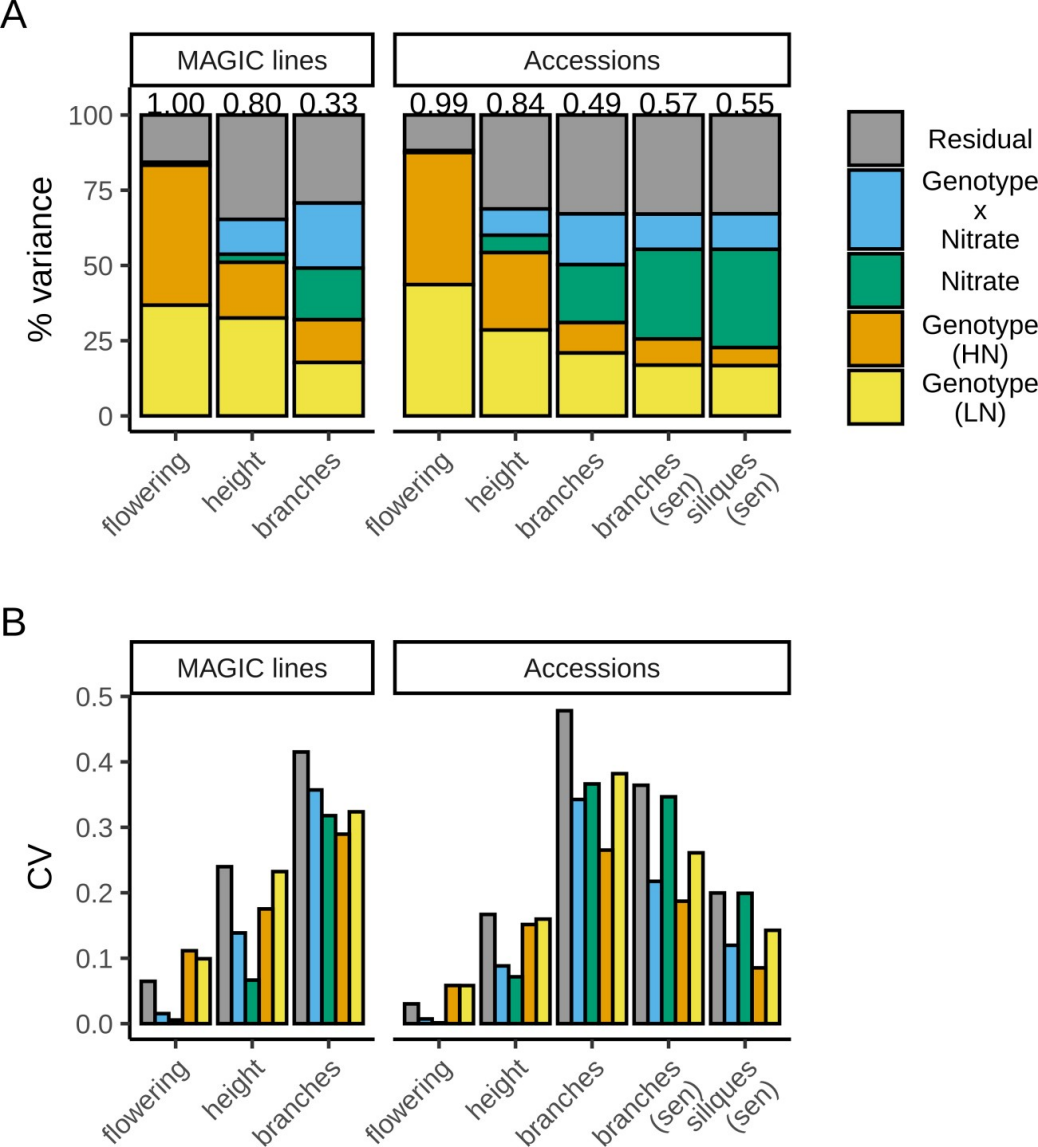
Genetic and non-genetic components of trait variation. Each trait’s variance was decomposed into genetic and non-genetic components using linear mixed models: genotype-specific effects both on high (HN) and low (LN) nitrate; nitrate-specific effects (environmental component); the response of each genotype to nitrate supply (genotype-by-environment interaction); and unexplained variance (residual). (A) shows the proportion of variance attributed to each component for each trait and (B) shows the magnitude of this variance relative to each trait’s mean, using the coefficient of variation (the estimated variance divided by the squared mean of the respective trait). Measurements of height and branch number were taken when the plants had two expanded siliques. For the accessions, the total number of branches was also scored at the senescence stage (sen), together with the total number of siliques. Variance components were estimated using data from 374 MAGIC lines and 297 (2 silique stage) or 278 (senescence stage) accessions with n = 4–8 replicates for each line on each nitrate treatment (median n = 8). Detailed results from these models are shown in S2 Table.

The effect of N treatment on the different traits was variable (Fig 1A). Most of the variation in flowering time was due to differences between genotypes (~83% together on LN and HN), with virtually no response to the N treatment (no plasticity). For height and shoot branching, the total genetic component of variance was lower, respectively ~52% and ~32%. However, for both of these traits, ~15% and ~39% of the trait variation respectively was related to the added effects of the nitrate environment and its interaction with the genotype (Fig 1A). Because of this, the response to N was variable across the MAGIC lines, with ~22% of the variation in shoot branching being attributable to GxE interaction. In fact, for this trait, the GxE component of variance was as large as the genetic component alone, with a significant contribution when compared with a reduced model that excludes it (Δ*AIC* = -419.32; likelihood ratio test p-value < 10^−6^); S2 Table), suggesting extensive genetic variation for shoot branching plasticity in these populations. Using these variance estimates from our models, we calculated the coefficients of variation for each trait’s component, to allow comparison among them (Fig 1B). This revealed that the largest relative variation was in shoot branching, whereas flowering time had comparatively little relative variation in our dataset. This is likely due to the fact that we worked primarily with rapid cycling lines that do not require vernalisation.

**Fig 2:**
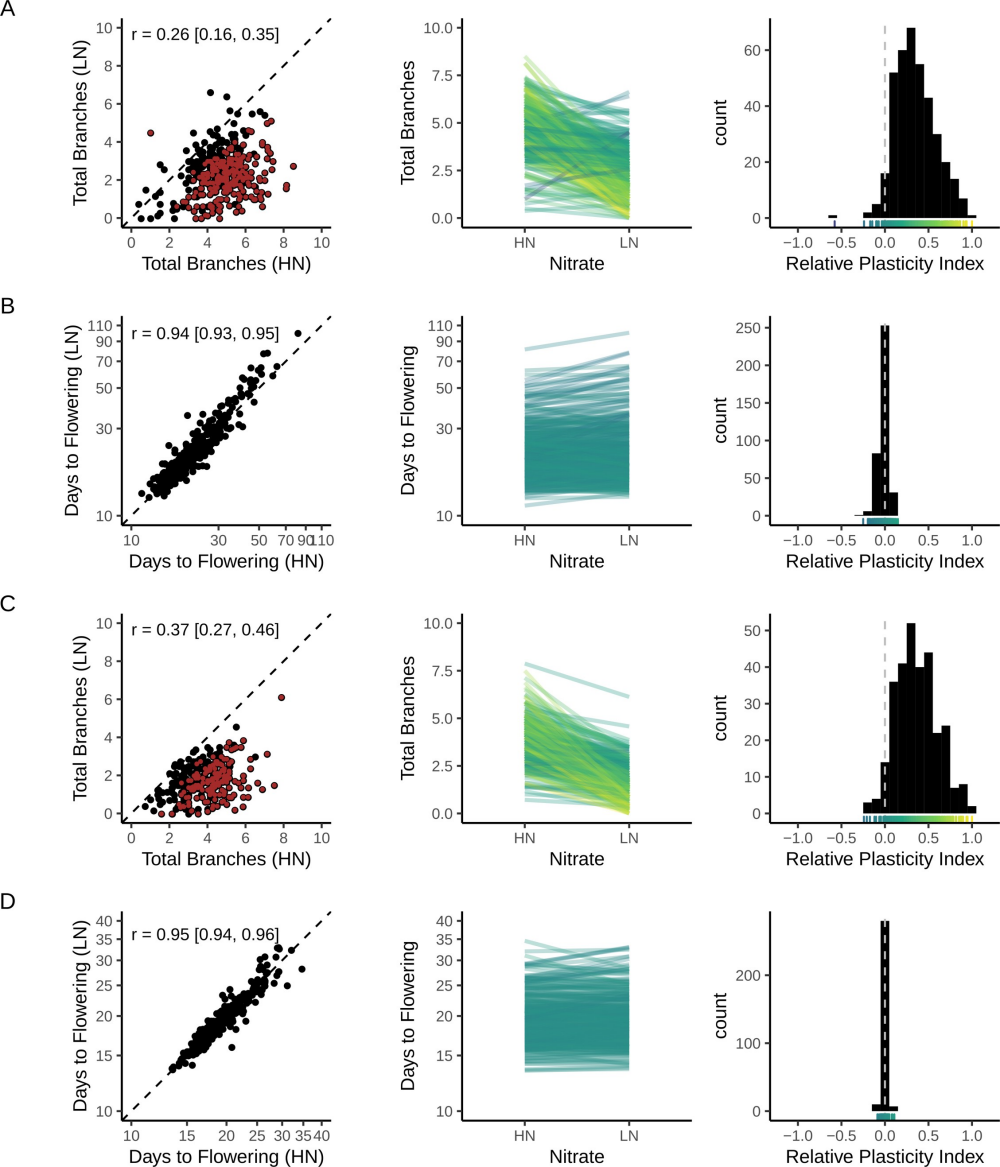
Relationship of trait means between the two nitrate treatments. Relationship between the mean trait value of each genotype grown on high (HN) or low (LN) nitrate supply shown as a scatter plot (left), as a reaction norm plot (middle) and as a histogram of relative plasticity (right). The latter shows the “relative distance plasticity index” of [105], which varies between -1 and 1 and with zero indicating no plasticity. Genotypes plotted in red on the left panel show significant plastic responses to N, as assessed by a Wilcoxon rank-sum test (false discovery rate < 5%). There were none for flowering time. The reaction norm plots are coloured by the relative plasticity index of each genotype, with the corresponding colour scale shown along the x-axis of the respective histograms. (A, B) Data from 374 MAGIC lines. (C, D) Data from 297 natural accessions. All plants were scored at the 2-silique stage, with means from n = 4–8 replicates per line in each nitrate treatment (median n = 8). Pearson’s correlation coefficient (r) is shown in each panel with the 95% confidence interval shown in brackets. The dashed line on the left panels is the identity line (x = y). Note the log-scale on the flowering time plots.

GxE interactions affect the overall variance of a trait in such a way that the mean trait value for a genotype in one environment is a bad predictor of the mean for that trait in another environment. For shoot branching, this can be readily seen as a low correlation between the number of branches for each line under the two nitrate treatments (Fig 2A), with an estimated genetic correlation of only ~0.33 (Fig 1A). By contrast, the flowering time for each line on HN and LN are very strongly correlated, as expected from the lack of plasticity we observed in this trait (Fig 2B), and the very high genetic correlation >0.99 (Fig 1A).

To assess whether natural accessions of Arabidopsis showed the same trends, 278 accessions were grown as described for the MAGIC lines. The same phenotypic traits were scored, but in addition the total number of secondary shoots was re-counted when the first siliques started to senesce, and the total number of siliques was scored as a proxy for reproductive fitness [17,32]. As expected, given the longer times available for branch growth, branch numbers on HN and LN were typically higher at senescence stage than at the 2-silique stage, with a strong positive correlation between the two developmental stages (S1 Fig). On both HN and LN, branch number correlated with silique number (S2 Fig), suggesting that branch number contributes to reproductive fitness, as observed in previous studies [15,17,33,34]. We note however that the number of seeds per silique can vary, associated with variation in silique length [35–38], which likely weakens this correlation between fruit number and seed number.

Similarly to the MAGIC lines, there was virtually no plasticity for flowering time, with the variation in this trait being primarily due to genotype (Fig 1). This lack of plasticity is reflected in the strong positive correlation between flowering time for each line on HN vs LN (Fig 2D). Also, as for the MAGIC lines, a substantial proportion of the variation in branch number and height is due to N supply, and ~17% and ~8% of the variation respectively is estimated to be due to GxE interaction (Fig 1). This GxE interaction results in weak correlation between branch numbers on the two N treatments (Fig 2C), with a significant GxE effect assessed by comparison with a reduced model without this component (Δ*AIC* = -237.95; likelihood ratio test p-value < 10^−6^); S2 Table). At the senescence stage, the variance was similarly partitioned for the number of branches and the number of siliques, consistent with the correlation between these two traits (S2C Fig).

### Shoot branching plasticity affects reproductive fitness both on high and low nitrate

**Fig 3:**
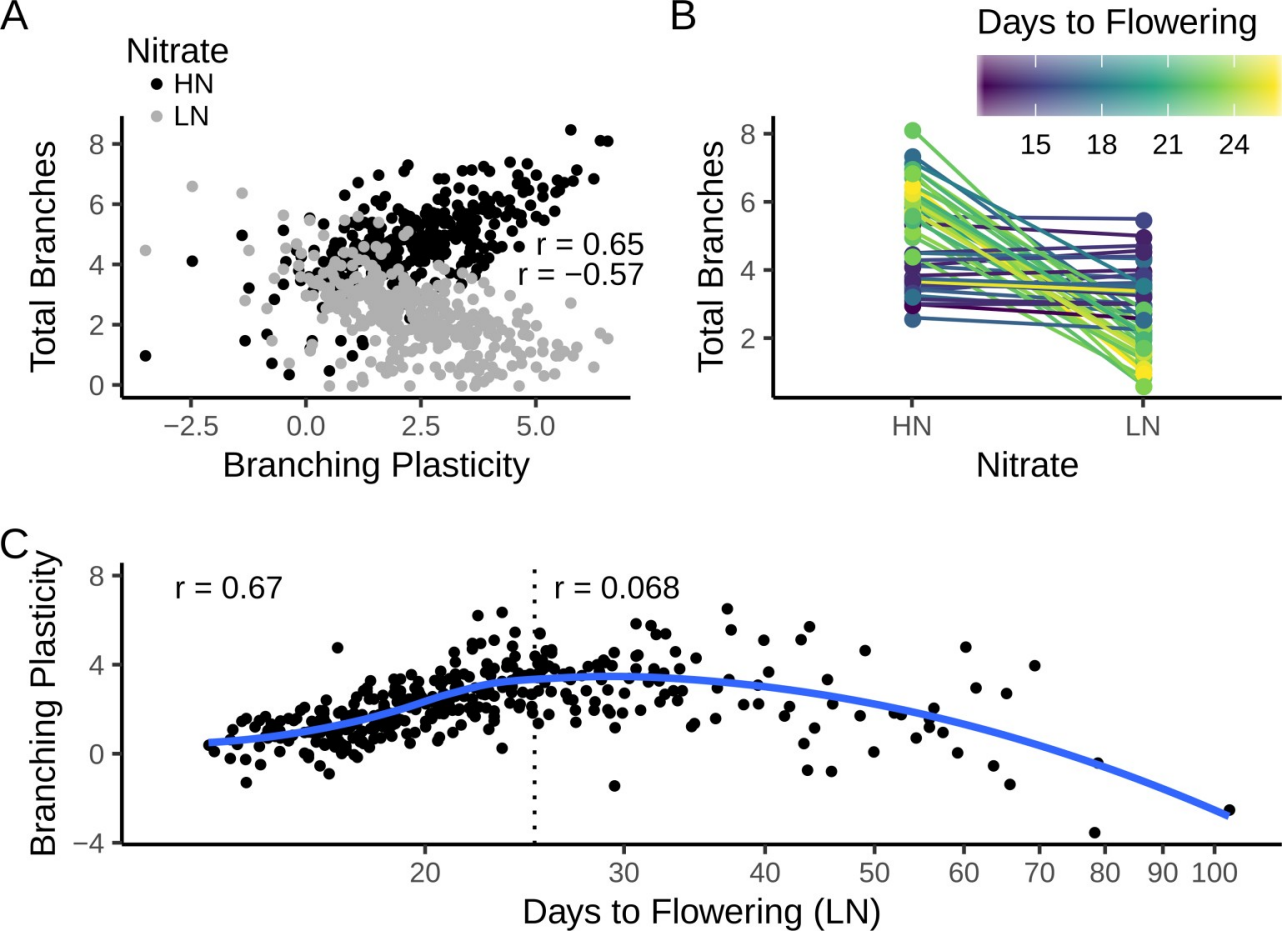
Trait correlations in the MAGIC lines. (A) Correlation between mean shoot branching plasticity and mean number of branches under low (LN) and high (HN) N supply. Pearson’s correlation coefficient (r) for each treatment is shown in the panels. The 95% confidence intervals are: r(LN) = -0.57 [-0.63, -0.49], p ~ 1^−33^; r(HN) = 0.65 [0.59, 0.71], p ~ 10^−46^. (B) Reaction norm plots of the number of branches for the 25 least and most plastic lines. Lines are coloured by the average days to flowering of each MAGIC line. (C) Correlation between mean days to flowering and branching plasticity. The blue line is a smoothed trend fitted by local regression (LOESS). The dotted line shows a cut-off of 25 days to flowering on LN. Pearson’s correlation coefficients are shown for all 374 lines (r = 0.068 [-0.033, 0.17], p = 0.19) and for 258 early-flowering lines only (r = 0.67 [0.60, 0.73], p ~ 1^−35^). In all panels, data are means from n = 4–8 replicates of each line (median n = 8). Note the log-scaled x axis on panel C.

**Fig 4:**
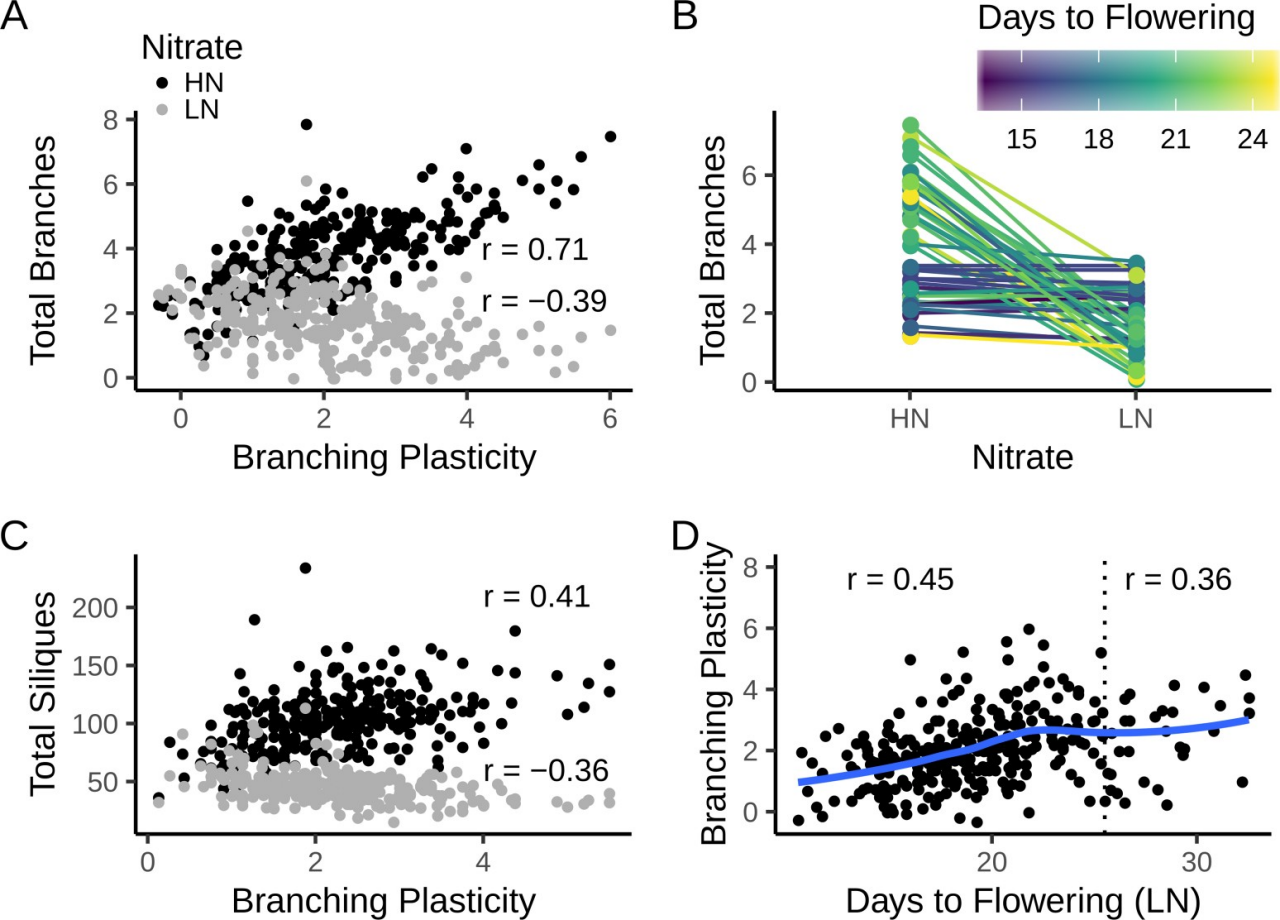
Trait correlations in the natural accessions. (A) Correlation between mean shoot branching plasticity and mean number of branches under low (LN) and high (HN) N supply at the 2-silique stage for 297 accessions. Pearson’s correlation coefficient (r) is shown for each nitrate treatment. The 95% confidence intervals are: r(LN) = -0.39 [-0.48, -0.29], p ~ 10^−12^; r(HN) = 0.71 [0.65, 0.76], p ~ 10^−47^; (B) Reaction norm plots of the number of branches for the 25 least and most plastic lines. Lines are coloured by the average days to flowering of each accession. (C) Correlation between mean shoot branching plasticity and total number of siliques produced at the senescence stage for 278 accessions. Pearson’s correlations and 95% CI: r(LN) = -0.36 [-0.46, -0.26], p ~ 10^−10^; r(HN) = 0.41 [0.31, 0.50], p ~ 10^−13^ (D) Correlation between mean days to flowering and branching plasticity. The blue line is a smoothed trend fitted by local regression (LOESS). The dotted line shows a cutoff of 25 days to flowering on LN. Pearson’s correlations are shown for all 297 accessions (r = 0.36 [0.26, 0.46], p ~ 10^−10^) and for 266 early-flowering accessions only (r = 0.45 [0.34, 0.54], p ~ 10^−14^). In all panels, data are means from n = 4–8 replicates of each accession (median n = 8).

To analyse shoot branching plasticity directly, we calculated the shoot branching response to N supply in each line as the difference between the mean number of secondary shoots formed on HN vs LN. Under HN conditions, for both the MAGIC lines (Fig 3A) and the natural accessions (Fig 4A), there was a strong positive correlation between total number of secondary shoots and shoot branching plasticity, whilst on LN there was a negative correlation between these traits. This can be clearly seen when plotting the mean branch numbers of the 25 most and least plastic lines from the two populations on HN and LN (Figs 3B and 4B). Genotypes that are highly branched on HN respond strongly to N deprivation, resulting in a very low branch number on LN; whilst less responsive lines typically have a moderate number of branches both on HN and LN. Thus, for shoot branching response to N, high plasticity is associated with phenotypic extremes, while low plasticity is associated with a constitutively intermediate phenotype. This contrasts with the phenotypes of shoot branching mutants where low plasticity locks plants into a constitutively a extreme phenotype [25,26].

As expected given the correlation between branch number and fruit number, when the natural accessions were grown on HN, there was a positive correlation between branching plasticity and silique number, whereas on LN the correlation was negative (Fig 4C). Thus the low plasticity lines have more fruits on LN, but the ability to protect branch numbers and hence fruit numbers on LN appears to come at the expense of the ability to exploit HN conditions by increasing branching and thus fruit set. Conversely, the high plasticity lines produce more siliques on HN.

### Shoot branching plasticity correlates with flowering time

We investigated the relationship between shoot branching plasticity and flowering time. Overall, there was a low or no significant linear correlation between shoot branching plasticity and flowering time in the two populations, although there is a clear non-linear trend in the data (Figs 3C and 4D). The trend seems strikingly linear for earlier flowering lines and to quantify this relationship, we excluded lines flowering after 25 days on LN, leaving 258 MAGIC lines, and 266 natural accessions. This was further justified by the fact that later flowering individuals showed some growth defects, especially on LN (e.g. stunted or aborted growth of the main stem and high levels of anthocyanin accumulation in the leaves). In both populations there is a strong positive correlation between the two traits (Figs 3C and 4D) with non-plastic lines flowering earlier than plastic lines. These data reveal a continuum, from lines that flower very early and produce a moderate number of branches regardless of N supply, to those that flower later and modulate their branch numbers according to N availability. The correlation is less strong in the natural accessions (compare Figs 3C and 4D), which in this experiment in general formed fewer branches, especially on LN.

These results suggest alternative strategies for growth under N limitation. At one extreme there is a rapid exit, escape strategy where plants flower early and branch regardless of N supply. This strategy results in higher fruit numbers on constitutively LN. At the other extreme are the later flowering lines that adjust their branching according to N supply. This strategy results in higher fruit numbers on constitutively HN. The lines we assessed form a continuum between these extremes.

### Shoot branching plasticity depends on shoot genotype

**Fig 5:**
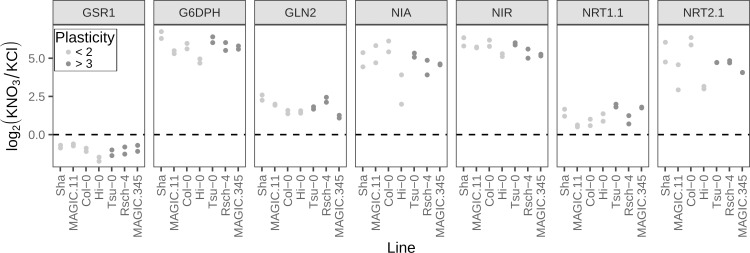
Expression of nitrate-responsive genes in low and high plasticity genotypes. Transcript levels were assessed by RT-qPCR in pooled seedlings growing in media in which nitrate was replaced by 0.5mM ammonium succinate; after 10 days the seedlings were treated with 5mM KNO_3_ or KCl for 2 hours and used for RNA extraction. Previous work has shown that *GSR1* is down-regulated in response to nitrate, whereas all the other genes are up-regulated [39,117,118]. The expression of each gene is shown as the log_2_(fold-change) between treatment (KNO_3_) and control (KCl) conditions (a value of zero indicates no difference between treatments—dashed line). The fold-change in expression was calculated using the “Delta Cp” (ΔΔCp) method [106] and normalised to two reference genes (APX3 and UBC9). Two biological replicates are plotted for each genotype. Genotypes are ordered by their branching plasticity (average branches on HN—LN) from the experiments detailed in Figs 1–4: Sha = 0.6, MAGIC.11 = 1.1, Col-0 = 1.4, Hi-0 = 1.6, Tsu-0 = 3, Rsch-4 = 3.8, MAGIC.345 = 6.4. None of the genes had a significant correlation between gene expression and shoot branching plasticity (in all cases the Spearman’s rank correlation p-value > = 0.2, bonferroni-adjusted for multiple testing).

The lack of response to N by lines at the low plasticity end of the spectrum could be due to an inability to sense nitrate. To test this hypothesis, we selected three lower plasticity lines (Sha and Hi-0 accessions and MAGIC.11) and three higher plasticity lines (Rsch-4 and Tsu-0 accessions and MAGIC.345) and assessed their primary nitrate response using a panel of seven nitrate responsive genes. Of these, six are up-regulated and one is down-regulated in response to nitrate resupply after a period of starvation (S3 Table; [39]). The standard lab accession, Col-0 (which has relatively low plasticity), was included for comparison. All the genes showed the expected nitrate response in all the lines, demonstrating that low plasticity lines are able to sense nitrate. For the genes tested, there was no clear correlation between the level of shoot branching plasticity and gene expression response to N supply (Fig 5).

**Fig 6:**
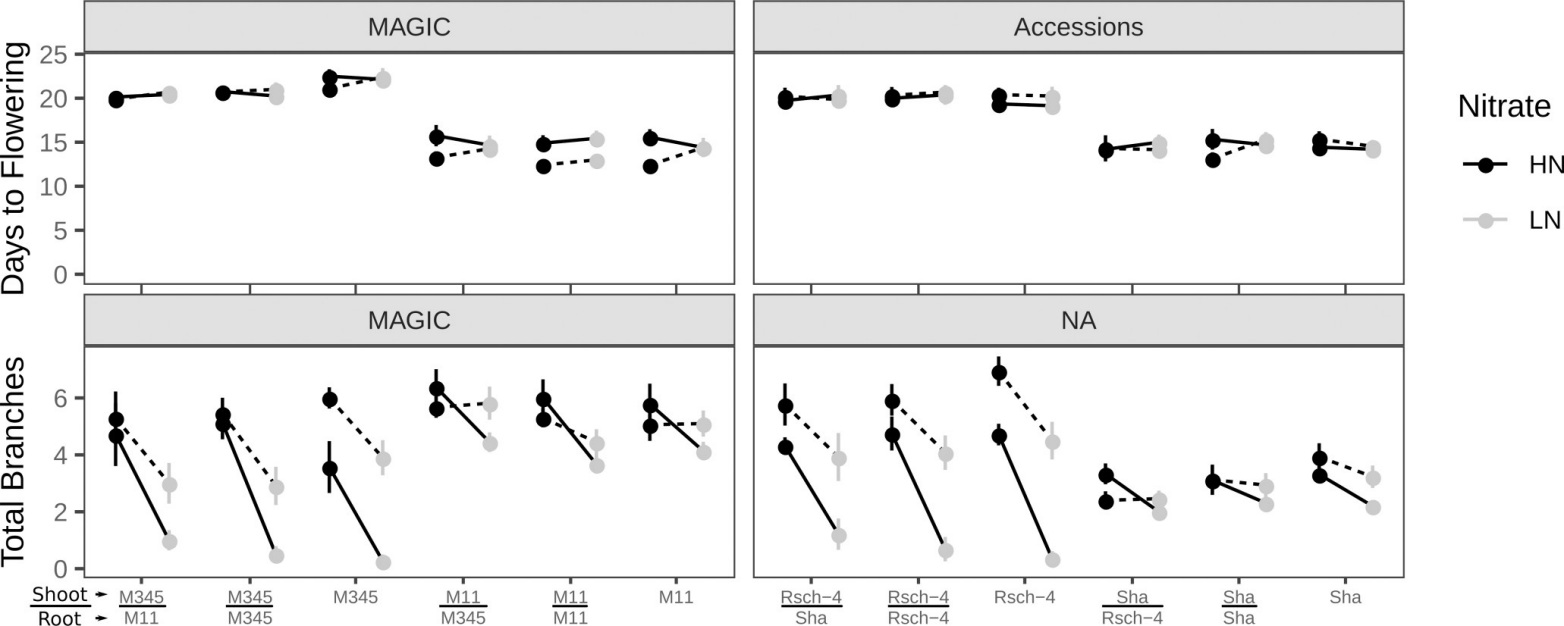
Grafting experiments between low and high plasticity lines. Mean shoot branching and days to flowering in reciprocal grafts between low plasticity (Sha accession and MAGIC.11) and high plasticity (Rsch-4 accession and MAGIC.345) lines. Data from two replicate experiments is shown (solid and dashed lines). We’ve tested the hypothesis of no effect of root and shoot genotypes as well as their interaction with the nitrate treatment (plasticity), for each trait using Wald F tests in a mixed model ANOVA (accounting for variation between experiments). For shoot branching there was a significant shoot-by-nitrate interaction [Wald F(1, 9.37) = 77; p ~ 7x10^-6^], after accounting for significant marginal effects of nitrate and experiment repeat. For flowering there was no significant organ-by-nitrate interaction (as expected from the lack of plasticity in this trait), but a significant marginal effect of shoot ideotype on the trait [Wald F(1, 9.10) = 264; p ~ 5x10^-8^]. There was no detectable effect of root or its interaction with nitrate. Data are means from n = 7–19 replicates per graft in each experiment (median n = 13); error bars are 2x standard error of the mean. Plants were scored at the 2-silique stage. Ungrafted and self-grafted plants are included as controls.

Rapid local transcriptional responses to nitrate are known to be distinct from plant N status responses [40–42]. We have previously shown that the shoot branching response to nitrate supply in Col is dependent on N status rather than nitrate per se [25]. Also, split root experiments have provided compelling evidence that N status assessment in Arabidopsis involves the shoot [41,42]. We therefore used reciprocal grafting experiments to assess whether the low plasticity and high plasticity syndromes were dependent on the shoot or root genotype. For the MAGIC lines, we used MAGIC.11 as a representative low plasticity line and MAGIC.345 as a high plasticity line. For the natural accessions, Sha (low plasticity) and Rsch-4 (high plasticity) were used. In both cases, the self-grafted and ungrafted controls reproduced the expected flowering time and plasticity phenotypes typical of low and high plasticity lines. In the grafts between genotypes, both the branching and flowering phenotypes were similar to those of the shoot parent (Fig 6). Thus, the shoot genotype determined the phenotype, with little evidence for any effect of root genotype on either flowering time or branching plasticity.

### Association mapping reveals common and distinct loci associated with flowering time and shoot branching

We next investigated the genetic architecture of shoot branching and its plasticity. Compared with the other traits we measured, shoot branching has generally lower broad-sense heritability, ranging from ~25–35% depending on the nitrate treatment and population analysed (S3 Fig). The heritability for flowering time was generally the highest (~70%), except for the subset of early-flowering MAGIC lines (<25 days on LN), where it was considerably lower (~25% on LN and ~35% on HN). This might be due to the fact that the underlying genetic variability in the MAGIC line population is much lower (with only 19 ancestors) than that in the accessions, and restricting the analysis to only the earliest flowering lines likely removed some of the large effect loci from the remaining population.

To identify QTL associated with each trait in the MAGIC lines, we focused on the early-flowering lines and used methods suitable for the analysis of multi-parent populations [43]. These methods differ from traditional bi-allelic SNP-based mapping methods, where the phenotype is associated with two genotypic classes, corresponding to the homozygous state of each allele. In the QTL mapping method used for MAGIC lines, the individuals’ genotypes at each locus are defined as probabilities that the allele derives by descent from each of the 19 founder accessions used to produce the mapping population [28]. This, therefore, is akin to a haplotype-based method of mapping, whereby the phenotype is associated with 19 possible genotypic states. In these MAGIC lines, variation in height is known to be largely due to the mutation in the *ERECTA* gene carried by one of the founding accessions, Ler-0 [28,44,45]. We could readily detect this QTL in our dataset, suggesting that the 258 lines used have sufficient power to detect associations with a simple genetic basis (S4 Fig).

**Fig 7:**
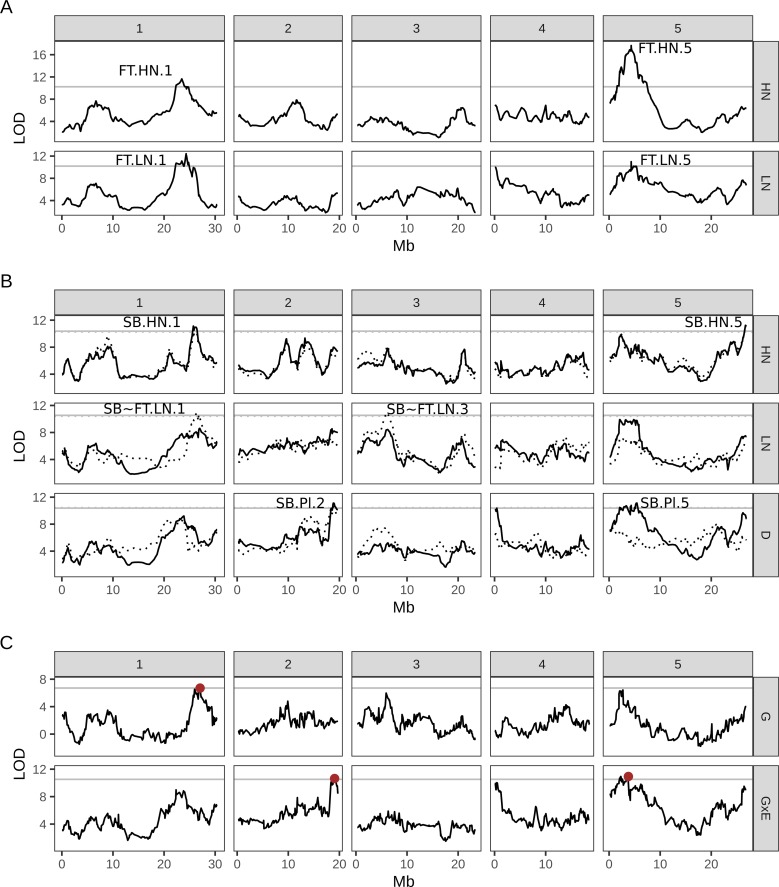
**QTL mapping for days to flowering (A) and shoot branching (B-C) in the MAGIC lines.** (A-B) QTL scans for each trait under high (HN) and low (LN) nitrate conditions and for shoot branching plasticity. To account for the correlation between shoot branching traits and flowering time, we also carried out the association test using flowering time as a covariate (dotted lines in B). (C) For shoot branching, we also fit a mixed model to the whole dataset simultaneously to separate common genetic (G) effects from gene-by-environment interaction (GxE) effects at each marker (this is somewhat equivalent to the plasticity QTL scan shown in panel B). In all panels, the plots show the LOD score of the association test carried out for each marker along the genome (see methods). The numbered panels correspond to each of the 5 chromosomes of Arabidopsis. The horizontal lines show the 5% genome-wide significance level based on 1000 permutations. Candidate QTL above this threshold are annotated for each trait and condition: SB, shoot branching; FT, flowering time; HN, high nitrate; LN, low nitrate; Pl, plasticity; SB~FT shoot branching including flowering time as a covariate. In panel C they are simply highlighted in red.

We also detected significant QTL for flowering time (Fig 7A), despite the lower heritability in this subset of lines when compared to the full set. There were two QTL coincident on HN and LN, both located in regions with genes previously implicated in flowering time regulation, such as *VIP5* and *FT* on chromosome 1 and, among others, *FLC*, *FY* and *CO* on chromosome 5 [46–48]. Together, these QTL explain ~10% of the phenotypic variance in this trait (S1 Table). Therefore, even among these early flowering lines, there is variation in flowering time that can be explained by these QTL.

We next mapped QTL for branch number under each N condition, as well as branching plasticity. Given the correlation between branching plasticity and flowering time (Fig 3C), we expected some QTL to co-locate with flowering time QTL. Indeed, a significant QTL was found on chromosome 5 for shoot branching plasticity, which coincided with a QTL for flowering time (SB.Pl.5 in Fig 7B). There was also a near-significant shoot branching plasticity QTL on the left arm of Chr4 that lies in the region of the flowering-related gene *FRI* (Fig 7B), which coincides with a non-significant peak for flowering time on LN (Fig 7A). These QTL are not significant when using flowering time as a covariate in the association model (Fig 7B, dotted line), suggesting that they are related to both traits, either epistatically or pleiotropically.

There was a QTL for shoot branching on high nitrate at the end of Chr5 (SB.HN.5 in Fig 7B), which remained even when using flowering time as a covariate in the model. This suggests a branching-specific association at this locus. We caution that this region of the chromosome suffers from poorer genotype imputations, with around half of the MAGIC line individuals having probability lower than 50% of assignment to a unique founder accession.

We also found a QTL specific for shoot branching plasticity on chromosome 2 (SB.Pl.2 in Fig 7B), which alone explains ~3% of the variance for shoot branching plasticity (S1 Table). To assess fully the independence of this GxE QTL from a common genetic effect across both nitrate treatments, we fitted the whole dataset simultaneously using a multi-trait model [20,49,50] (Fig 7C). Comparing the likelihood of models with and without an interaction term between nitrate and the genotype at each marker suggests that the QTL on chromosome 2 specifically controls GxE variation for shoot branching, but not common genetic effects across both nitrate treatments. Similarly, consistent with the covariate analysis, this model identifies the QTL on Chr5 that is related to the correlation with flowering time.

Interestingly, when using flowering time as a covariate in the QTL mapping, two additional cryptic QTL for branch number on low N were identified (SB~FT.LN.1 and SB~FT.LN.3 in Fig 7B). We note that even though the QTL on Chr1 seems to coincide with the one for flowering time (FT.HN.1 and FT.LN.1 in Fig 7A), the peak SNP for this QTL is located ~2.4Mb away from it, suggesting separate loci in this region are associated with each trait. In fact SB~FT.LN.1 coincides with SB.HN.1 and indeed this QTL is captured in the joint model (Fig 7C), suggesting a common effect in both nitrate treatments. Together, all the QTL identified for this trait explain ~10% of the trait’s added genetic and GxE variance (S1 Table).

**Fig 8:**
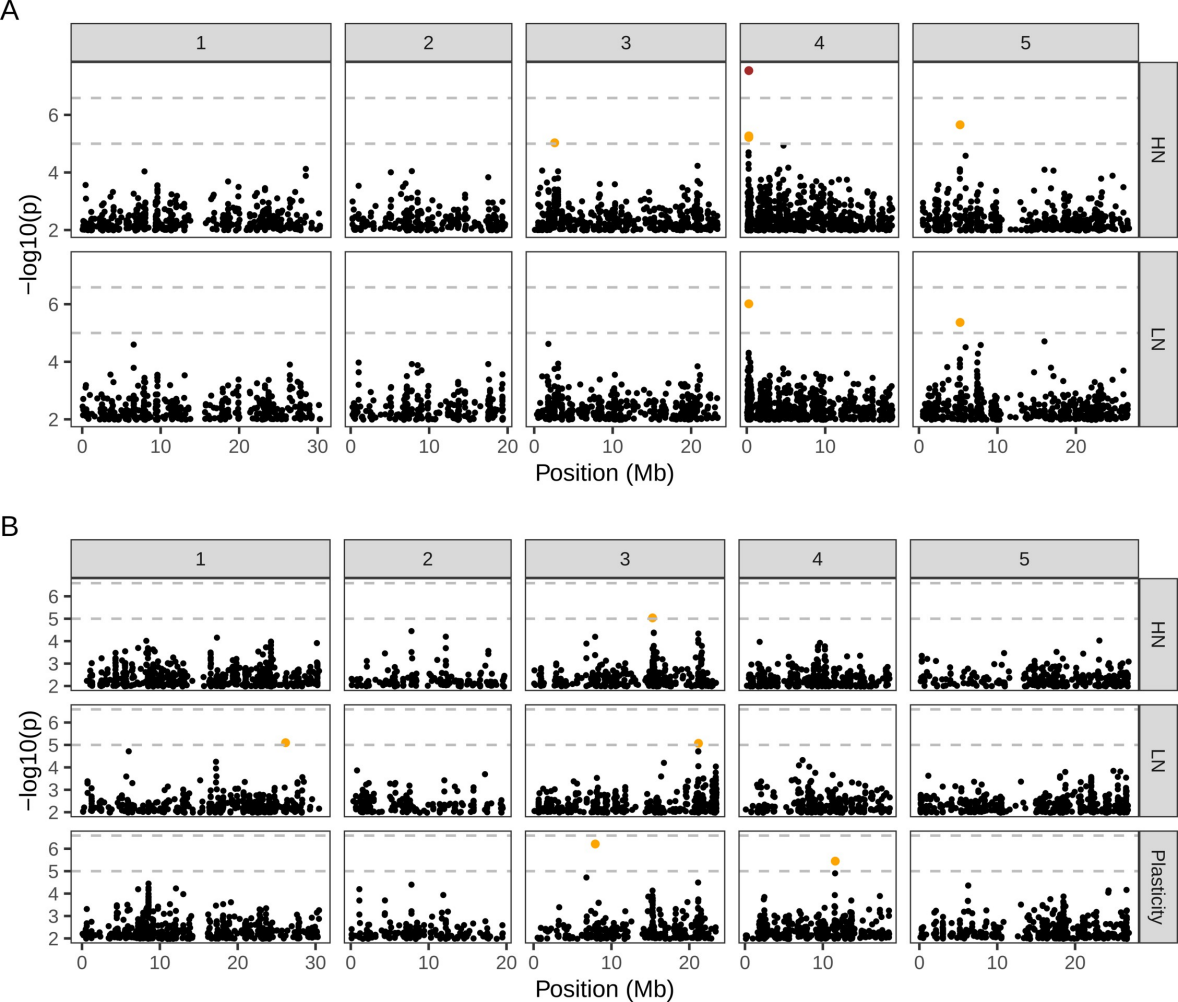
**QTL mapping for days to flowering (A) and shoot branching (B) in accessions.** Manhattan plots showing the association mapping results for each trait on high (HN) and low (LN) nitrate. For shoot branching, QTL mapping was also performed for this trait’s plasticity. The upper horizontal dashed line is the 5% genome-wide significance threshold, obtained with bonferroni correction; one SNP above this threshold is shown in red. The lower line, at p = 10^−5^, was defined based on the inclusion of two known QTL for flowering time; SNPs above this relaxed threshold are shown in orange. The association test was carried out using a linear mixed model that corrects for population structure by taking into account the genetic relatedness between individuals [114]. The tests used 192863 bi-allelic SNPs that had >5% frequency in our sample of 240 early flowering accessions scored at 2-silique stage (flowering <25 days on LN).

To explore whether similar loci could be identified in the natural accessions, we performed association mapping in 240 of the natural accessions used in the phenotyping experiments for which genotypes are available for 192 863 bi-allelic SNPs with >5% frequency [51]. Across all the traits, we found only one significant QTL for flowering time on HN in a region of Chr4, which has also been reported in other GWA studies for flowering time (Fig 8; [48,52]).

Similarly to the MAGIC lines, we fitted shoot branching data from both nitrate treatments simultaneously using a multi-trait model to test for effects common across both nitrate treatments as well as GxE effects (S5 Fig) [20]. The result revealed a single SNP passing the 5% genome-wide threshold for a common genetic effect on the trait. The SNP was located on Chr5, between genes AT5G20680 and AT5G20670, both of unknown function. Generally, other SNPs neighbouring this SNP had high likelihood for no association, making it unclear whether this is a true or spurious association, as a correlated signal would be expected in neighbouring SNPs due to linkage (for example, the two closest SNPs within 1Kb had p ~ 0.5 and p ~ 0.8). A spurious association could be due to the fact that this SNP had relatively low minor allele frequency of 6.5%. Furthermore, no QTL were identified using a multi-SNP approach, which takes advantage of increased power to detect associations based on local additive association signals around a focal window of the genome (S6 Fig) [53]. Overall, this suggests a low power to detect associations in our dataset.

To investigate whether the failure to identify any QTL for the remaining traits was due to a lack of markers in linkage disequilibrium with causal loci, we estimated the heritability of each trait using a SNP-based relatedness matrix (h^2^_GWAS_; [54]), thus assessing the variance jointly explained by all markers used in the association test (S7 Fig). For flowering time h^2^_GWAS_ ~ 0.9, suggesting that our SNP panel captures most of the phenotypic variance for this trait, but this variance cannot be pinpointed to individual SNP loci (a case of “hidden” heritability). Shoot branching h^2^_GWAS_ estimates were lower than our broad-sense heritability estimates, with the exception of branches at the senescence stage on HN. The apparent increase in h^2^_GWAS_ for this trait between the 2-silique and senescence stage could be due to higher variability within genotypes at the 2-silique stage than at the senescence stage. The generally low h^2^_GWAS_ values suggest that the markers do not capture the genetic component of the variance in this trait (a case of “missing” heritability). One explanation for this is that the markers used here are not in linkage disequilibrium with the causal loci. However, we also calculated h^2^_GWAS_ using a panel of ~1.7M imputed SNPs, which did not improve the result, suggesting this “missing” heritability might be due to other complexities of the genetic architecture of this trait that cannot be captured by h^2^_GWAS_.

If shoot branching is a polygenic trait, our GWAS might be severely under-powered to detect low-effect loci due to the relatively low number of accessions used (240), when compared with other studies (nowadays on the order of one thousand [55]). Furthermore, Bonferroni-corrected thresholds are often over-conservative resulting in a high number of false negative results [56–58]. Therefore, in order to identify suggestive QTL, we defined a new genome-wide threshold by taking advantage of prior knowledge of flowering QTL on Chr4 (near the CCT gene) and Chr5 (near the FLC gene) [20,55,59]. This new threshold, at p < 10^−5^, allowed inclusion of these two flowering QTL on both LN and HN (orange points in Fig 8A). With this relaxed threshold we found 5 suggestive QTL for shoot branching (orange points in Fig 8B). Two of these were in the vicinity of QTL found in the MAGIC lines: the SNP on Chr1 for LN is 2Mb away from QTL “SB~FT.LN.1”; the SNP on Chr3 for Plasticity is ~180Kb away from QTL “SB~FT.LN.3”. This suggests that the new threshold might be picking biologically significant loci, and these tentative associations further confirm the hypothesis that shoot branching and its plasticity are complex polygenic traits.

### Allelic heterogeneity in the MAGIC line QTL

**Fig 9:**
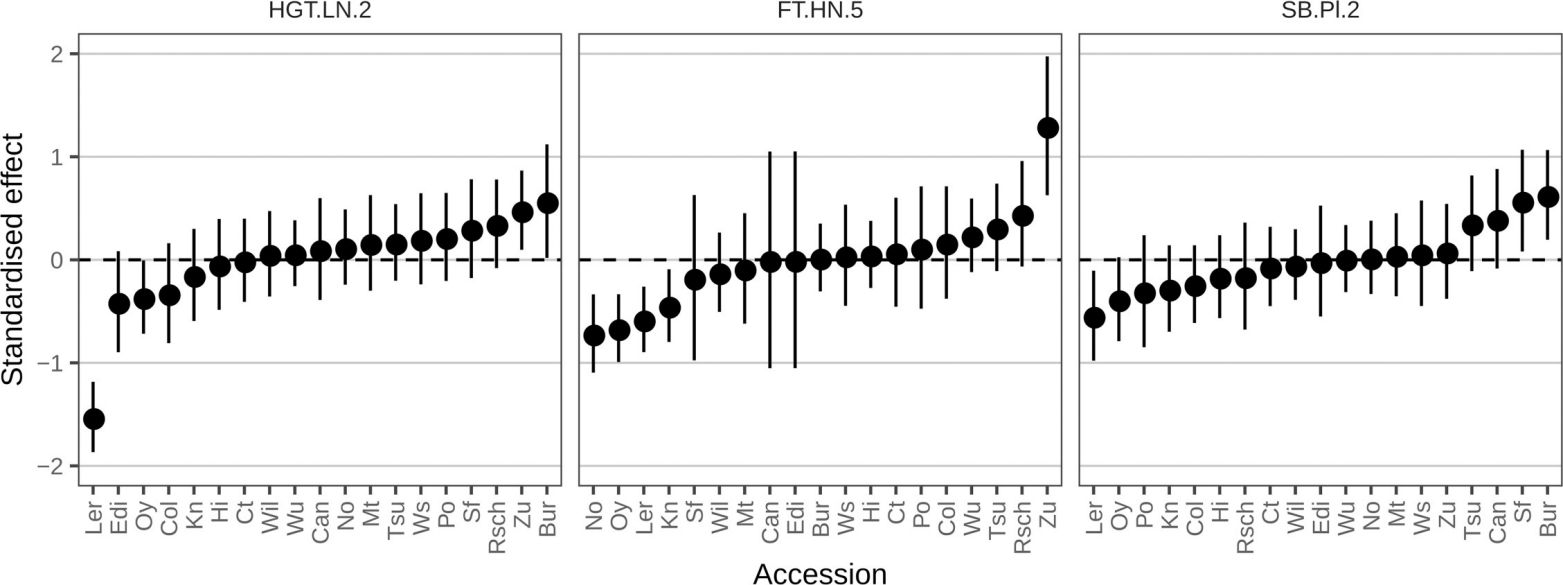
Predicted parental effects for QTL identified in this work. The QTL are named as in S4 Fig, Fig 7 and S1 Table. The points and error bars are the mean and 95% confidence intervals of the Best Linear Unbiased Predictors (BLUPs) of the 19 haplotype effects estimated using the R/*qtl2* package [43]. The y-axis shows the standardized QTL effect (i.e. the values indicate how many standard deviation units each estimate deviates from the trait’s mean).

Another hypothesis to explain the failure of GWAS to find significant associations is that multiple alleles at a single locus might be associated with the trait of interest [20,51,60,61]. Because this allelic heterogeneity is not captured by bi-allelic SNP markers, it could lead to a failure to detect QTL. The MAGIC lines provide a good system to explore this allelic heterogeneity, because it is possible to infer the phenotypic effect of each parental genotype at a significant QTL from the phenotypic mean of MAGIC lines inferred to carry a particular parental haplotype at that locus [28]. For example, as mentioned above, the height QTL on chromosome 2 is known to be related to the large-effect null allele carried by the Landsberg *erecta* (Ler) accession, which is apparent in the predicted allelic effects on height at this locus in the MAGIC population (Fig 9). Besides the large effect L*er* allele, there is further allelic heterogeneity captured in these MAGIC lines, which might be due to subtle effects of other alleles. Similarly, the Zu allele at FT.HN.5 has a major effect on delaying flowering time compared to the other parents, again with substantial variation in the effect of the remaining alleles of around 1 standard deviation both above and below the respective mean. By contrast, the predicted effects of the parental alleles at the plasticity QTL detected on Chr2 (SB.Pl.2) do not show a clear large effect allele, but rather a range of lower size effects within 1 standard deviation of the overall mean. This is consistent with a more subtle effect of multiple alleles at the locus affecting shoot branching plasticity, which might not be captured with simpler bi-allelic genotype associations.

## Discussion

Theoretical analyses of the circumstances under which developmental plasticity is adaptive identify a number of important factors that influence the balance between the costs and benefits of plastic responses [2,5–7,62]. These include the spatiotemporal scales of environmental heterogeneity compared to the spatiotemporal scales over which plastic responses occur and the costs and benefits of those responses. Here we use shoot branching plasticity in response to N supply as a model system to investigate natural genetic variation in plasticity. This system is of interest because N availability in nature is known to vary extensively over short spatiotemporal scales [63,64] relative to branching responses; while the high costs of producing additional branches are balanced by the potential for high benefits through additional fruits and seeds. Thus costly shoot branching investment decisions must be made ahead of reliable information about the future availability of N.

Optimal foraging strategy for N has been studied extensively in roots (reviewed in [65]). There is evidence to support sophisticated risk-benefit calculations underpinning root plasticity. For example, pea plants in which the root systems have been divided between two chambers will proliferate lateral roots into a chamber where the N supply is highly variable only if the supply to the other half of the root system, which was kept constant, was low [66]. While these studies demonstrate correlations between root behaviour and shoot biomass, plastic responses to nutrient supply in the shoot are in general less well characterised.

Our results demonstrate that in natural accessions of Arabidopsis, and in a collection of MAGIC lines derived from 19 natural accessions, shoot branching plasticity correlates positively with flowering time and fruit set on high nitrate, but negatively with fruit set on low nitrate. In contrast, flowering time was relatively insensitive to N supply. Our results are comparable to those observed in a series of studies in Arabidopsis by Pigliucci and Schlichting, who found extensive variation in plasticity for shoot branching and other traits in response to nutrient levels [22,23,67]. In particular, a study of 37 families derived from three populations grown under high and low nutrient conditions showed similar trends to our findings, namely low plasticity for flowering time and variable plasticity for branching and height between families [67]. The relatively high branching of the low plasticity lines on low N, coupled with their earlier flowering can be interpreted as a rapid exit strategy in response to nutrient limitation. In contrast, the late flowering and low branching phenotype of the high plasticity lines on low N may represent an N foraging strategy [68]. Given the potentially high spatiotemporal heterogeneity of nitrate in soil [63,64], late flowering extends the time over which this nutrient could be encountered and captured by the plant. Indeed, late flowering has also been implicated as a phosphate foraging strategy [69].

One framework to interpret these data is to consider the relationship between phenotypic plasticity and the evolution of specialist and generalist lifestyles. Often, generalist species, which occupy a wide range of habitats, are associated with high plasticity (e.g. invasive species [6,70], although this may not be a general feature [9,10]). On the other hand, specialist species may be associated with extreme, stable environments, where low plasticity and extreme phenotypes might evolve. A well studied case is that of the shade avoidance response in plants. In many species, shading by neighbouring plants triggers stem elongation and a suite of other responses supporting shade avoidance in a highly competitive environment [71]. While in some species shading by neighbouring plants triggers stem elongation and a suite of other plastic responses supporting shade avoidance, in other species that are adapted to shaded environments there is a lack of such response. For the shoot branching syndromes we identify, non-plastic lines could be considered as specialised for stably N-deficient soils, whereas plastic lines would be able to exploit a range of environments with variable N availability. However, rather than having an extreme branching phenotype, the low plasticity lines maintain a moderate branching phenotype on both high and low N, while the highly plastic genotypes make very few branches on low N and many branches on high N. Thus, in contrast to the shade avoidance example, for natural genetic variation in the shoot branching response to N supply, high and low branching extremes are associated with high plasticity.

Across the two populations, there is a continuum of responses between the low and high plasticity extremes, correlating with flowering time. This could reflect a tradeoff between investing existing resources in the next generation and gathering more [3,8,72]. In this context it is interesting that the ability to protect branching under low N appears to come at the expense of the ability to exploit high N, despite the developmental potential to do so. This suggests general N insensitivity. However, all the lines we tested are able to sense nitrate, as indicated by changes in transcript abundance for six nitrate responsive genes following N supply. This is similar to what is reported in other studies, in which variation in ability to response to N limitation was associated with only small changes in nitrate-responsive gene expression [73,74]. Consistent with this idea, we have previously shown that shoot branching responds to N sources other than nitrate and could therefore be a response to plant N status, in which shoot N plays an important role [25]. In this context it is interesting that reciprocal grafting experiments demonstrated that both the “low plasticity; early flowering” and “high plasticity; late flowering” phenotypes are determined by the shoot genotype. This contrasts to the branching phenotype of both strigolactone and cytokinin biosynthetic mutants, where wild-type roots can restore wild-type shoot branching to hormone deficient shoots [26,75,76]. This argues against the phenotypes of low or high plasticity lines being caused by constitutive changes in strigolactone or cytokinin biosynthetic capacity. Consistent with this interpretation, we have previously shown that low branching on low N is dependent on the plant hormones auxin and strigolactone [25], while high branching on high N requires a third hormone, cytokinin [26]. Double mutants defective in strigolactone and auxin synthesis/signalling are constitutively highly branched, whereas mutants defective in cytokinin synthesis/signalling show constitutively low levels of branching regardless of N supply. This contrasts with the moderate levels of branching associated with low plasticity in the populations we examine here. Nonetheless, since both strigolactone and cytokinin are synthesised throughout the plant, and their levels can be modulated by nutrient supply [77,78], a shoot specific effect on hormone synthesis linked to N status is possible. In this context it is interesting that the *MAX3* strigolactone biosynthetic gene is located near the peak of the QTL for shoot branching plasticity detected in the MAGIC lines (SB.PL.2 in Fig 7).

In general, despite the fact that the shoot branching and flowering time traits we measured had substantial levels of heritability, we recovered relatively little of the variation in our mapping experiments. In the GWA analysis of the natural accessions, no SNPs significantly associated with any of the branching traits were identified and for flowering time only one SNP was identified in the region of the well-known *FRIGIDA* locus [48,52]. Even considering our stringent threshold for association significance, these results suggest that either there are many loci of small effect involved (e.g. [56,79]), and/or there are multiple alleles at each locus (e.g. [20,51,60]). Both of these genetic complexities affect the statistical power to detect associations, in particular in complex populations such as the ones used here [56,80]. Although we found some tentative associations when relaxing our significance threshold (based on prior knowledge of flowering time QTL), we caution that these may incur high false discovery rates and would require confirmation in studies with larger populations, or other advanced methods of association (e.g. genomic prediction and/or multi-marker methods [56,81]).

Although our approach has the advantage of including a wide range of the natural genetic diversity in Arabidopsis, providing a broad picture of shoot branching GxE in this species, it comes at the expense of a lower statistical power compared to traditional bi-parental populations. Studies using bi-parental RIL populations are often able to identify many QTL for growth and physiological traits under different environmental conditions including N supply [82–84]. For example, a recent study using 4 Arabidopsis RIL populations under different water availability treatments was able to reveal a complex genetic architecture for several growth-related traits, which are, by their very nature, integrative of several developmental events in a plant’s life [85]. The results revealed several associations exclusive to only one of the RIL populations, suggesting that these QTL may not have been detected in traditional GWAS. Therefore, one possible way to dissect further the genetics of shoot branching GxE is to produce a RIL population specifically between a plastic and a non-plastic accessions.

Consistent with this idea, mapping in the accessions was less effective than in the MAGIC lines, where there are fewer parental haplotypes involved [28,80,86]. For many of the loci where significant associations were detected in the MAGIC population, there was evidence for variable effects of the parental haplotypes on the traits, suggestive of allelic heterogeneity for some of these loci.

Despite the relatively few loci identified, the mapping results from the MAGIC lines reveal some interesting features. First, there are significant peaks for shoot branching that are not significant or even clearly detectable for shoot branching plasticity. For example, two QTL for branch number on low N, SB~FT.LN.3 and SB~FT.LN.1 (Fig 7B), were detected when using flowering time as a covariate in the QTL model. There is no evidence of an effect of these loci on branching plasticity despite branch number on low N being inversely correlated with plasticity. The *BRC1* and *BRC2* genes, which have been implicated in branching and its plasticity lie within these two regions, respectively [87–89]. Conversely, there are peaks for branching plasticity that are not apparent when branch number is mapped, such as SB.Pl.2 (Fig 7B and 7C). Together these data suggest that branch number and its plasticity can be tuned at least partially independently.

Several significant peaks for flowering time were detected in regions of the genome known to include major flowering time regulators. In some but not all cases, these correlated with significant peaks for shoot branching traits, for example FT.HN.5 and SB.Pl.5 (Fig 7). The flowering time regions detected all include genes involved in season detection, such as *FT*, *FLC* and *FRI* [47,90]. This suggests the interesting possibility that branching plasticity may be seasonally controlled, with variation at these loci independently underlying variation in flowering time and plasticity. Indeed, a previous study using the outbred population from which the Arabidopsis MAGICs were derived revealed a pleiotropic role for the *FRI* gene, which besides controlling flowering time also affected the number of inflorescence nodes and associated branches of plants carrying recessive non-functional alleles [33]. This effect was dependent on *FLC* genotype, which is expected from the epistatic interaction between these two loci [91].

It is important to note that the lines we studied all flower rapidly, so the effects we see are primarily of relevance to a rapid-cycling lifestyle. *FRI* and *FLC* are typically studied in the context of vernalization requirement, so it is interesting that they may also contribute to flowering time in these early flowering lines. Although under our conditions flowering was not plastic, this trait is sensitive to seasonal and temperature changes [20,90,92]. It will therefore be interesting to understand how environmentally-induced changes in flowering interact with the branching architecture of plants and their response to nitrate.

Our findings might also be of agronomic relevance, where breeding for increased nitrogen use efficiency is of importance [93]. There are several QTL studies looking at this issue in a range of crop species (e.g. in rice [94,95], wheat [96,97], barley [98,99], sorghum [100], maize [101]). Although the specific traits analysed vary across studies, the broad picture that emerges is the polygenic nature of yield-related traits, with dozens of candidate loci found across studies, often with GxE effects related to N availability. In this context, our study emphasises the importance of GxE in understanding the genetic architecture of such traits. Further, it illustrates that breeding efforts under non-limiting nitrate conditions might result in worse performing genotypes when fertilization is reduced. In fact, studies in maize have shown that yield improvements under low N conditions are lower if the cultivars were selected on high N, rather than directly on LN [93,102]. Dissecting the mechanisms behind these differences remains a challenge [93], and our study in a model organism may help to address some of these questions in the future.

Overall, our work identifies intriguing associations between branching, its plasticity and flowering time, which may have adaptive significance. Our analysis suggests the hypothesis that a rapid escape strategy combining early flowering with uncoupling of shoot N status from branching suppression allows seed yield to be maintained in N-poor environments. This may provide tools to understand better shoot N status sensing, which is currently enigmatic. However, the genetic complexity of the natural variation we have identified suggests that selected bi-parental mapping populations may be more powerful in determining the underlying genetic basis for these traits and their association than the multi-accession approaches we used here.

## Materials and methods

### Plant material

We used two *Arabidopsis thaliana* populations for our experiments: 297 natural accessions and 374 MAGIC (Multiparent Advanced Generation Inter-Cross) lines. The MAGIC lines are derived from 19 natural accessions that were randomly inter-crossed for 4 generations, followed by 6 generations of self-fertilisation to generate inbred lines, typically used for QTL mapping [28]. The natural accessions were obtained from the Nottingham Arabidopsis Stock Centre (NASC, www.arabidopsis.info accessed Dec 2018) and were selected from several collections [29–31]. Only accessions described to have a flowering time of less than 55 days were included in the experiments (data from [31,48]; Arabidopsis Biological Resource Center, http://abrc.osu.edu accessed Dec 2018; own experimental data).

### Growth conditions

For each line, seeds were sown on wet filter paper and stratified for five days at 5°C in the dark, and then transferred to 5.5 cm diameter pots filled with low nitrate substrates consisting of 50% sand (Leighton Buzzard sand from WBB Minerals) and 50% Terragreen (Oil-Dri). The substrates were wetted with *Arabidopsis thaliana* salts (ATS) solution [103], containing either 9mM (high N treatment, HN) or 1.8 mM NO_3_^-^ (low N treatment, LN), which we have previously shown represent N-sufficient and N-deficient conditions for Col-0 [25]. After two weeks, plants were fed once a week with 10ml of nutrient solution per pot, and in-between watered with regular tap water as needed. In all experiments plants were grown under glasshouse conditions. For the QTL experiments plants were grown in the summers of 2008 (MAGIC lines) and 2012 (accessions). For each line, eight replicates were grown on each nitrate treatment, which were randomly allocated to trays around the glasshouse.

### Trait measurements

For our main natural variation experiments we measured flowering time, total branches and height for each plant. Flowering time was measured as the number of days from germination to the day at which the first flower buds were visible at the rosette centre. Total branches were counted as the number of secondary shoots (from the axils of rosette + cauline leaves) that were more than ~1cm in length. Height was measured as the length of main inflorescence stem.

Total branches and height measurements were made when plants had formed two full siliques (2-silique stage). For 278 of the 297 accessions, we also obtained measurements at a later stage when plants had at least two senescing siliques (senescence stage). Traits are reported as averages for each line, with each line represented by 4–8 replicate plants (median n = 8).

Branching plasticity was calculated for each line as the difference between the mean number of secondary shoots formed on HN vs LN. This measure was chosen taking into consideration the biology of shoot branching. It may seem attractive to normalise the number of branches to the number of nodes, and thus the total number of possible branches. Similarly, branching plasticity could be expressed as a proportional change in the number of branches. These measures were rejected based on the fact that branching occurs in a strict basipetal sequence and nitrate supply modulates the stopping point of that sequence [25,104]. Since branch activation at any one node is therefore highly dependent on its position along the primary axis and on the behaviour of the buds at more apical nodes, proportional measures of branch activity are inappropriate. Node number does provide the upper bound for primary branch number, but this is seldom achieved except in extreme branching mutants.

To allow plasticity comparisons across traits, we’ve also calculated a relative metric, the “relative distance plasticity index” adapted from [105]. For each line, we calculated all pairwise differences (between replicates) of the trait value on HN and LN and then divided it by the respective pairwise sum of those values. This division ensures the plasticity measure is unitless, allowing comparisons across traits on different scales. These pairwise scaled plasticity differences were then summed and divided by the number of pairwise comparisons, to get an average scaled plasticity. This results in a metric that varies between -1 and 1, with zero indicating no plasticity.

### Primary nitrate response

The expression of primary nitrate responsive genes was analyzed by RT-qPCR in four natural accessions (Shahdara, Hi-0, Rsch-4 and Tsu-0), two MAGIC lines (MAGIC.11 and MAGIC.345) and the standard laboratory line Col-0. For each sample, 10mg of seeds were surface sterilized and stratified for 4 days at 5°C and then transferred to 25ml of liquid ATS in which nitrate was replaced by 0.5mM ammonium succinate. The seeds were left to germinate and grow on a shaker (100-120rpm) in a controlled environment room (16h light/8h dark, 17–21°C). After 10 days, the seedlings were treated with 5mM KNO_3_ or KCl for 2 hours. After this treatment, they were quickly dried and flash frozen in liquid nitrogen, and stored at -80°C until RNA extraction. Total RNA was extracted using the RNeasy Plant mini kit including DNase I treatment (Qiagen), following the manufacturer’s instructions. RNA was quantified using a NanoDrop 1000 and 1 μg was used to produce cDNA using Superscript II (Invitrogen), following the manufacturer’s instructions. qPCR reactions were prepared using LightCycler 480 SYBR Green I Master (Roche), with 5ng of cDNA in 20μl reactions, following the manufacturer’s instructions. Reactions were performed in a LightCycler 480 II (Roche) machine and C_p_ values were determined based on the “second-derivative maximum” method implemented in the manufacturer’s software.

There were two biological replicates for each line, with three technical replicates each. The C_p_ values of the technical replicates were averaged for each biological replicate and used in subsequent calculations. For each sample, the transcript levels of the primary nitrate response genes were normalised relative to the mean C_p_ value of two reference genes: APX3 (AT4G35000) and UBC9 (AT4G27960). Finally, we estimated the relative expression of those genes in the treatment (KNO_3_) relative to the control (KCl) conditions. These estimates were made using the ΔΔC_p_ method, assuming equal primer efficiency [106]. All primers are listed in S3 Table.

### Grafting

Two pairs of lines were used for grafting: two MAGIC lines (MAGIC.11 and MAGIC.345) and two accessions (Shahdara and Rsch-4). For each of these pairs we made four pairwise grafting combinations: two autografts and two allografts (one in each direction). Plants were germinated on ATS medium containing 0.8% bacto-agar and either 9mM or 1.8mM of NO_3_^-^. From thereon, the grafting experiment was performed as described in [26]. Flowering time and total branches were measured at silique stage as described above. 7 to 19 replicates (median n = 13) were sown for each graft combination, along with the ungrafted parents and the whole experiment was replicated twice.

The lines used in these experiments represented the two ideotypes of focus: low plasticity and early flowering (MAGIC.11 and Shahdara) and high plasticity and later flowering (MAGIC.345 and Rsch-4). To assess whether each ideotype’s characteristic phenotype was mainly shoot or root driven, we tested the hypothesis of no effect of root and shoot ideotypes on each trait using a mixed model ANOVA. This model included fixed terms for nitrate treatment, shoot ideotype, root ideotype and experiment (this was included as a fixed rather than random term since there were only two levels for this factor). We further included interaction terms between nitrate and each of the root and shoot ideotypes to account for their contribution to the trait’s plasticity (ideotype-by-nitrate interaction). Finally, we included a random term for each graft’s ID (to account for variation in the base level, or intercept, of the trait for each particular graft combination) and a random slopes term for nitrate (to account for the specific plasticity of each graft combination). The mixed model was fitted with the statistical program R [107], using the *lmer* function of the *lme4* package [108]. The hypothesis of no effect of root and/or shoot ideotypes along with the interaction of these terms and nitrate were tested using Wald F tests as implemented in the *Anova* function in the *car* R package [109].

### Heritability and variance partitioning

We estimated broad-sense heritabilities based on replicate measurements of each genetic line. Strictly speaking, this is a measure of “clonal repeatability”, but in a randomized experiment like ours it should give a good estimate of the degree of genetic determination of the trait, i.e. its broad-sense heritability (p. 123 in [110]). Briefly, we used linear mixed models to partition the phenotypic variance into between-line (genetic) and within-line (residual) components. Broad-sense heritability was calculated by dividing the genetic variance by the total variance estimated from the model. Heritability estimates were obtained separately for each population and nitrate treatment. Confidence intervals for these estimates were obtained using a parametric bootstrap approach [111]. In summary, we simulated 1000 sets of phenotype data based on the fitted model and estimated broad-sense heritability for each. A 95% confidence interval was obtained by taking the 0.025 and 0.975 quantiles of the heritability distribution of simulated phenotypes.

To partition the variance into genotype, environment and genotype-by-environment (interaction) components, we fitted a more complex random slopes linear mixed model [112]. We included nitrate treatment as a fixed effect, genotype ID as a random effect and a random slopes term for the nitrate-by-genotype interaction. In more detail, the GxE mixed model fit to each trait was

$$Y_{ij}=\beta_{0}+\beta_{1}NITRATE+u_{0j}+u_{1j}+\epsilon_{ij}$$

where: ***Y***_***ij***_ is the trait value for the i-th individual from the j-th genotype (MAGIC line or accession ID); ***β***_***0***_ is the intercept of the model, which in our specification is the mean on LN; ***β***_***1***_ is the response when on HN; ***NITRATE*** is a dummy variable indicating whether the individual was grown on HN; ***u***_***0j***_ is the random term for varying intercepts of the j-th genotype (genotype-specific average on LN); ***u***_***1j***_ is the random term for varying slopes of the j-th genotype (the genotype-specific response to nitrate, or GxE component); ϵ_**ij**_ is the residual term with $\epsilon ∼Normal(0,\sigma_{e}^{2})$. The random part of the model has the following variance-covariance specification:

$$\left[\begin{array}{c} u_{0j}\\ u_{1j} \end{array}\right]∼Normal(0,\Omega_{u})\;\Omega_{u}=\left[\begin{array}{cc} \sigma_{u0}^{2} & \\ \sigma_{u01} & \sigma_{u1}^{2} \end{array}\right]$$

Where: σ^2^_u0_ is the variance of the trait on low nitrate; σ^2^_u1_ is the variance of the trait responses on HN (the GxE component); σ_u01_ is the covariance between the two. The covariance parameter was used to calculate the correlation of the trait values between LN and HN. The estimates from the model are presented in S2 Table for each trait. We also compared this full model with a reduced model that excluded the GxE component: *Y_ij_* = *β*_0_ + *β*_1_*NITRATE* + *u*_0*j*_ + *ϵ_ij_*. We assessed differences between the models using a likelihood ratio test (to obtain a p-value) and difference in the Akaike Information Criterion (where negative values indicate a loss of information in favour of the more complex model).

In all cases, linear mixed models were fitted with *R* [107], using the *lmer* function of the *lme4* package [108] and variance components were extracted using custom scripts. Flowering time data were log-transformed and total siliques data were square-root-transformed to reduce distributional skews and heteroskedascitity, thus improving the model’s diagnostics. Despite the fact that shoot branching is measured on a discrete scale (count data), we did not observe a strong relationship between the mean and variance across samples as is expected with count data, typically modelled using Poisson likelihood models. For this reason, we modelled our branching data using a normal likelihood function assuming homogeneous variance.

### Association mapping in MAGIC lines

Association (QTL) mapping in the MAGIC lines was performed using the *R/qtl2* package [43] and a custom R data package containing the genotype data in a suitable format for analysis (available at https://github.com/tavareshugo/atMAGIC). In summary, for each of the 1254 available markers, the probability of ancestry of an individual’s genotype at that marker was inferred using the function qtl2::calc_genoprob(), assuming a 1% genotyping probability error. The qtl2::scan1() function was then used to fit the QTL model to each marker for each trait analysed. For shoot branching, we also fitted a model that included flowering time as a covariate (to account for the correlation between these traits). We used a 5% genome-wide significance threshold obtained by permutation using the qtl2::scan1perm() function. The founder accession’s effect at each candidate QTL was estimated using the qtl2::scan1blup() function following [43]. These analysis used the average trait value for each line in each nitrate treatment (n = 4–8 replicates each).

For shoot branching, we also fitted a more complex “multi-trait” model, to assess the independence of GxE QTL from common effect QTL [20,49,50]. This model was similar to the variance partitioning model detailed above (a random slopes mixed model), but with an added term to account for the genotype of each MAGIC line. Due to the model complexity, we converted the founder genotype probabilities obtained from *R/qtl2* to a single genotype value corresponding to the founder allele with maximum probability at each marker for each individual (i.e. the genotype variable was a factor with 19 levels, corresponding to each founder accession). LOD scores were obtained for two model contrasts: the full model compared to a genetic model with no genotype-by-nitrate interaction (GxE) term; the genetic model compared to a null model (no genotype term). We obtained a 5% genome-wide threshold by permutation of the genotype data. This analysis was done using custom *R* scripts (see data availability section).

Finally, we estimated the variance explained by each marker by comparing the genetic variance using the mixed models just described with a model excluding the QTL marker as a predictor variable, similarly to [113].

### Association mapping in accessions

Association (GWAS) mapping in the accessions was performed using the “--mlma-loco” function in GCTA 1.26.0 [114]. This performs an association test for each SNP using a linear mixed model that includes a random term to account for population structure. This is achieved by using a SNP-based relatedness matrix to model the variance-covariance structure between genotypes in the population. We excluded the marker being tested from the relatedness matrix using the “leaving-one-chromosome-out” (LOCO) method implemented in GCTA, which should increase the power to detect associations. SNP genotypes for accessions were obtained from the 250K dataset of [51] (available at http://github.com/Gregor-Mendel-Institute/atpolydb, last accessed Jul 2019), which were converted to plink format (www.cog-genomics.org/plink/1.9/formats, last accessed Jul 2019) using a custom python script. SNPs with minor allele frequency below 5% were discarded, leaving 192863 biallelic SNPs. A 5% genome-wide significance threshold was obtained by Bonferroni correction. The “--reml” function in GCTA 1.26.0 was used to estimate the proportion of phenotypic variance explained by the SNPs used in the GWAS, referred to as GWAS heritability, *h*^*2*^_*GWAS*_.

We also estimated *h*^*2*^_*GWAS*_ using a panel of 1 763 004 imputed SNP genotypes provided by Ümit Seren in the group of Magnus Nordborg. This is the set of imputed SNP genotypes used in the web application “GWA-Portal” (http://gwas.gmi.oeaw.ac.at, last accessed Jul 2019).

For shoot branching, we also fitted a “multi-trait” model using the *limix 0*.*7*.*12* Python package [115]. Similarly to what was done with the MAGIC lines, this was used to test for genetic effects common to both nitrate treatments as well as GxE effects (following [20]). Finally, we also obtained “multi-SNP” associations using sets of SNPs within 10Kbp windows centered on each gene’s annotation, using the “--fastBAT” method in GCTA 1.26.0 [53]. In all cases genome-wide thresholds were obtained by bonferroni correction.

### Statistical analysis

Data analysis and visualisation were carried out using the statistical software R version 3.4.1 [107]. The meta-package *tidyverse* [116] was used for data manipulation and visualisation. Where relevant, approximate 95% confidence intervals for mean estimates are presented as 2x standard error of the mean (assuming data follow a normal distribution). Other specific analysis or statistical tests are described in the relevant sections above or in figure legends. All analysis scripts are provided with the supplementary data (see Data availability statement), but are also available with detailed information at: https://github.com/tavareshugo/publication_deJong2019_Nplasticity

## Supporting information

S1 Fig Relationship of branch number between developmental stages. Data are shown separately for plants growing on high (HN, left) or low (LN, right) nitrate. Data are means from 278 accessions from n = 4–8 replicates per line in each nitrate treatment (median n = 8). Pearson’s correlation coefficient (r) is shown in each panel with the 95% confidence interval shown in brackets (in all cases p < 10^−6^). The dashed line is the identity line (x = y).
(TIFF)

S2 Fig Relationship between number of branches and fertility traits. Correlation between seed weight and total branches (A) or total siliques (B) at senescence in a set of 4 MAGIC lines (cross symbols) and 7 accessions (dot symbols). Data are means of n = 12–15 replicates per line (error bars show 2x standard error of the means). (C) Correlation between number of branches and number of siliques at senescence stage in 278 accessions. Data are means of n = 4–8 replicates per line (median n = 8). Pearson’s correlation coefficient (r) is shown in each panel with the 95% confidence interval shown in brackets (in all cases p < 10^−6^).
(TIFF)

S3 Fig Broad-sense heritabilities for traits in each N treatment. The top panels show the heritability for all measured lines in each population: 374 MAGIC lines and 297 accessions scored at 2-silique stage; 278 accessions scored at senescence (sen). The bottom panels show estimates for the subset of early-flowering lines (mean flowering time <25 days on LN): 258 MAGIC lines and 266 accessions scored at 2-silique stage; 260 accessions at senescence. Error bars are the 95% confidence interval of the estimate obtained by a bootstrap procedure (see methods). For each line and each nitrate treatment n = 4–8 (median n = 8).
(TIFF)

S4 Fig QTL mapping for height in the MAGIC lines. The plot shows the LOD score of the association test carried out for each marker along the genome (see methods). The numbered panels correspond to each of the 5 chromosomes of Arabidopsis. The horizontal dashed line shows a 95% genome-wide threshold based on 1000 permutations. Candidate QTL above this threshold are annotated for each condition: HGT, height; HN, high nitrate; LN, low nitrate. The major QTL on chromosome 2 coincides with the *Erecta* gene. The QTL on chromosome 5 co-localises with QTL for flowering time (Fig 7A), and is due to a positive correlation between these two traits (Pearson’s r = 0.55, 95% CI [0.46, 0.63]; p = 10^−16^).
(TIFF)

S5 Fig Multi-trait QTL mapping for shoot branching in accessions. Manhattan plots showing the association mapping results of a multi-trait model simultaneously modelling shoot branching variation on high (HN) and low (LN) nitrate. Two model contrasts were performed to test for a genetic effect common to both nitrate treatments (G, upper panel) and an interaction GxE effect (GxE, bottom panel). The horizontal dashed lines show the 5% genome-wide significance threshold, obtained with bonferroni correction.
(TIFF)

S6 Fig **Multi-SNP QTL mapping for (A) flowering time and (B) shoot branching in accessions**. Manhattan plots showing the results of a set-based test that used the results from the test shown in Fig 8, to produce joint summary statistics for sets of SNPs contained within 10Kbp windows centered on each annotated Arabidopsis gene [53]. The horizontal dashed lines show the 5% genome-wide thresholds, obtained by Bonferroni correction.
(TIFF)

S7 Fig Heritability estimates based on GWAS SNPs. Heritability estimates based on the genetic relatedness between samples, inferred from all SNPs used in the GWAS for 240 accessions, h^2^_GWAS_ [54]. h^2^_GWAS_ is estimated from a linear mixed model that regresses the phenotype to a genetic relatedness matrix, arriving at an estimate of the variance explained by it. h^2^_GWAS_ is thus the proportion of total phenotypic variance that can be attributed to variance in the relatedness between individuals. h^2^_GWAS_ was estimated based on two sets of SNPs: 192 853 SNPs from [51] (upper panel); a set of 1 763 004 imputed SNP genotypes (kindly provided by Magnus Nordborg) (lower panel). Error bars show the standard error of the estimate, based on 240 accessions at 2-silique stage and 234 accessions at senescence stage.
(TIFF)

S1 Table Summary of significant QTL in MAGIC lines. The location of each QTL peak is given in relation to the Arabidopsis TAIR10 reference genome. For each peak marker we give the LOD score and an estimate of the explained variance at that QTL (methods, [113]). Where several QTL were identified for the same trait, they were also fitted at once to obtain a joint estimate of explained QTL variance.
(PDF)

S2 Table Detailed results of mixed effects models used to partition trait variances into genetic and non-genetic components. The model specification is detailed in the methods. For each trait, model estimates are shown for the fixed and random parts of the model. Fixed terms: “*LN mean”* is the intercept of the model (*β*_*0*_); *“Plasticity mean”* is the mean response to HN across lines (*β*_*1*_). Random terms: “*LN*” is the variance of the trait on low nitrate (σ^*2*^_*u0*_); “GxE” is the variance of the trait responses to HN (the GxE component, *σ*^*2*^_*u1*_); “*ρ(LN;GxE)*” is the correlation between the trait on LN and its response to HN; “*ρ(LN;HN)*” is the correlation of the trait on LN and HN. Both correlations were calculated from the variance-covariance estimates. The full model was compared to a reduced model that excluded the GxE component: *Y_ij_* = *β*_0_ + *β*_1_*N* + *u*_0*j*_ + *ϵ_ij_*. We assessed differences between the models using a likelihood ratio test (to obtain a p-value) and changes in the Akaike Information Criterion, “Δ*AIC*” (where negative values indicate a loss of information in favour of the more complex model). To allow easier comparison across traits, their values were centered on the mean and scaled to the standard deviation.
(PDF)

S3 Table Primer sequences of genes used in RT-qPCR of nitrate-responsive genes. (PDF)
