# Supplementary material for: Natural variation in Arabidopsis shoot branching plasticity in response to nitrate supply affects fitness
Source: PLoS Genet. 2019 Sep 20;15(9):e1008366. doi: 10.1371/journal.pgen.1008366 (PMC6774567; doi:10.1371/journal.pgen.1008366)
Supplement: S1 Table — The location of each QTL peak is given in relation to the Arabidopsis TAIR10 reference genome. For each peak marker we give the LOD score and an estimate of the explained variance at that QTL (methods, [113]). Where several QTL were identified for the same trait, they were also fitted at once to obtain a joint estimate of explained QTL variance. (PDF) [file pgen.1008366.s008.pdf]

Trait	Nitrate	QTL ID	Peak SNP ID	Chr	bp	LOD	Genetic variance explained (%)	Joint genetic variance explained [b] (%)
Flowering time	High	FT.HN.1	MN1_23474588	1	23,470,918	11.6	3.71	9.41
		FT.HN.5	MN5_4327715	5	4,327,712	17.6	6.75	
	Low	FT.LN.1	MN1_24322296	1	24,318,694	12.4	3.94	
		FT.LN.5	MN5_4327715	5	4,327,712	11.0	2.65	
Shoot branching	High	SB.HN.1	MASC00557	1	25,735,094	11.1	2.96	9.82
		SB.HN.5	MN5_26708459	5	26,691,233	11.2	3.60	
	Low	SB~FT.LN.1	MN1_26278413	1	26,274,750	10.8	1.99	
		SB~FT.LN.3	MN3_5910420	3	5,910,414	10.9	2.28	
	Plasticity	SB.PL.2	SOC1_461	2	18,811,694	11.1	3.11[a]	
		SB.PL.5	MN5_5452600	5	5,452,597	11.1	2.77[a]	

[a] For the plasticity trait the genetic variance refers to the proportion of GxE variance from a multi-trait model (Fig 7C, methods).

[b] The joint genetic variance was calculated from a multi-trait model as the proportion of the added within-nitrate variance plus the GxE (genetic plasticity) variance components.
