# Supplementary material for: Natural variation in Arabidopsis shoot branching plasticity in response to nitrate supply affects fitness
Source: PLoS Genet. 2019 Sep 20;15(9):e1008366. doi: 10.1371/journal.pgen.1008366 (PMC6774567; doi:10.1371/journal.pgen.1008366)
Supplement: S3 Table — (PDF) [file pgen.1008366.s010.pdf]

**Gene name (TAIR ID)**	**Forward primer (5' --> 3')**	**Reverse primer (5' --> 3')**	**Nitrate response (reference)**
UBC9 (AT4G27960)	TGTACAAGACAGACAAGAACAAGTACGA	TGTGTCAGCCCATGGCATA	reference gene
APX3 (AT4G35000)	GCCGTGAGCTCCGTTCTCT	TCGTGCCATGCCAATCG	reference gene
NRT1.1 (AT1G12110)	GTGGCCTCCTCCTAACCACC	CATAGACGAATGGCGACGC	upregulated [36]
NRT2.1 (AT1G08090)	GGGCTAACGTGGATGGGAGT	GCAAACCGGAGGCTTCCTTG	upregulated [36]
NIR1 (AT2G15620)	TGCTTAACACGAGGCGAGG	GTACACGTCAGCACCCTCGA	upregulated [36]
NIA1 (AT1G77760)	CGGTAATCGCCGCAAGGAAC	TAGCCGGATCCATCGCCATC	upregulated [36]
G6PD3 (AT1G24280)	CTACGCTTCTCCTCAGGGGC	ACGGACCTCCTCTGCGATC	upregulated [36]
GLN2 (AT5G35630)	ACACTCCTGTGGGAGCCAAC	GCTTTTGAGCTGCAAGGGC	upregulated [98]
GLN1-1/GSR1 (AT5G37600)	GTGCTGGTGCTCACTGCAAC	GGTTCGCAACACCCCAAAGG	downregulated [97]
