# Supplementary material for: Natural variation in Arabidopsis shoot branching plasticity in response to nitrate supply affects fitness
Source: PLoS Genet. 2019 Sep 20;15(9):e1008366. doi: 10.1371/journal.pgen.1008366 (PMC6774567; doi:10.1371/journal.pgen.1008366)
Supplement: S2 Table — The model specification is detailed in the methods. For each trait, model estimates are shown for the fixed and random parts of the model. Fixed terms: “LN mean” is the intercept of the model (β0); “Plasticity mean” is the mean response to HN across lines (β1). Random terms: “LN” is the variance of the trait on low nitrate (σ2u0); “GxE” is the variance of the trait responses to HN (the GxE component, σ2u1); “ρ(LN;GxE)” is the correlation between the trait on LN and its response to HN; “ρ(LN;HN)” is the correlation of the trait on LN and HN. Both correlations were calculated from the variance-covariance estimates. The full model was compared to a reduced model that excluded the GxE component: Yij = β0 + β1N + u0j + ϵij. We assessed differences between the models using a likelihood ratio test (to obtain a p-value) and changes in the Akaike Information Criterion, “ΔAIC” (where negative values indicate a loss of information in favour of the more complex model). To allow easier comparison across traits, their values were centered on the mean and scaled to the standard deviation. (PDF) [file pgen.1008366.s009.pdf]

Fixed terms	Accessions					MAGIC		
	**Flowering**	**Height**	**Branches**	**Branches (sen)**	**Siliques (sen)**	**Flowering**	**Height**	**Branches**
LN mean	0.08	-0.33	-0.55	-0.64	-0.66	0.06	-0.21	-0.53
Plasticity mean	-0.04	0.61	1.07	1.26	1.3	-0.09	0.41	1.05
Variances and correlations from random effects (percent of total variance in parenthesis)								
LN	0.86 (44)	0.41 (27)	0.15 (13)	0.12 (12)	0.08 (9)	0.81 (47)	0.29 (19)	0.23 (17)
HN	0.85 (44)	0.46 (30)	0.31 (26)	0.22 (24)	0.22 (25)	0.64 (37)	0.52 (33)	0.29 (21)
GxE	0.01 (1)	0.14 (9)	0.25 (21)	0.16 (17)	0.15 (18)	0.02 (1)	0.18 (12)	0.35 (26)
Residual	0.23 (12)	0.5 (33)	0.49 (41)	0.44 (47)	0.43 (49)	0.27 (16)	0.55 (36)	0.47 (35)
ρ(LN;GxE)	-0.08	-0.2	-0.23	-0.17	-0.06	-0.82	0.08	-0.52
ρ(LN;HN)	0.99	0.84	0.49	0.57	0.55	1	0.8	0.33
Comparison with reduced model (without GxE)								
ΔAIC	-0.58	-81.73	-237.95	-133.13	-155.81	-43.18	-166.33	-419.32
p-value	0.11	$< 10^{-6}$	$< 10^{-6}$	$< 10^{-6}$	$< 10^{-6}$	$< 10^{-6}$	$< 10^{-6}$	$< 10^{-6}$
